# Supplementary material for: The molecular basis of monopolin recruitment to the kinetochore
Source: Chromosoma. 2019 Apr 30;128(3):331–54. doi: 10.1007/s00412-019-00700-0 (PMC6823300; doi:10.1007/s00412-019-00700-0)
Supplement: Supplementary file 1 — (DOCX 6.84 kb) [file 412_2019_700_MOESM1_ESM.docx]

**Supplementary Information for Plowman, Singh *et al.*, 2018**

Figs. S1-S11

Tables S1-S5

Movies S1-S9

Supplementary sequences

**
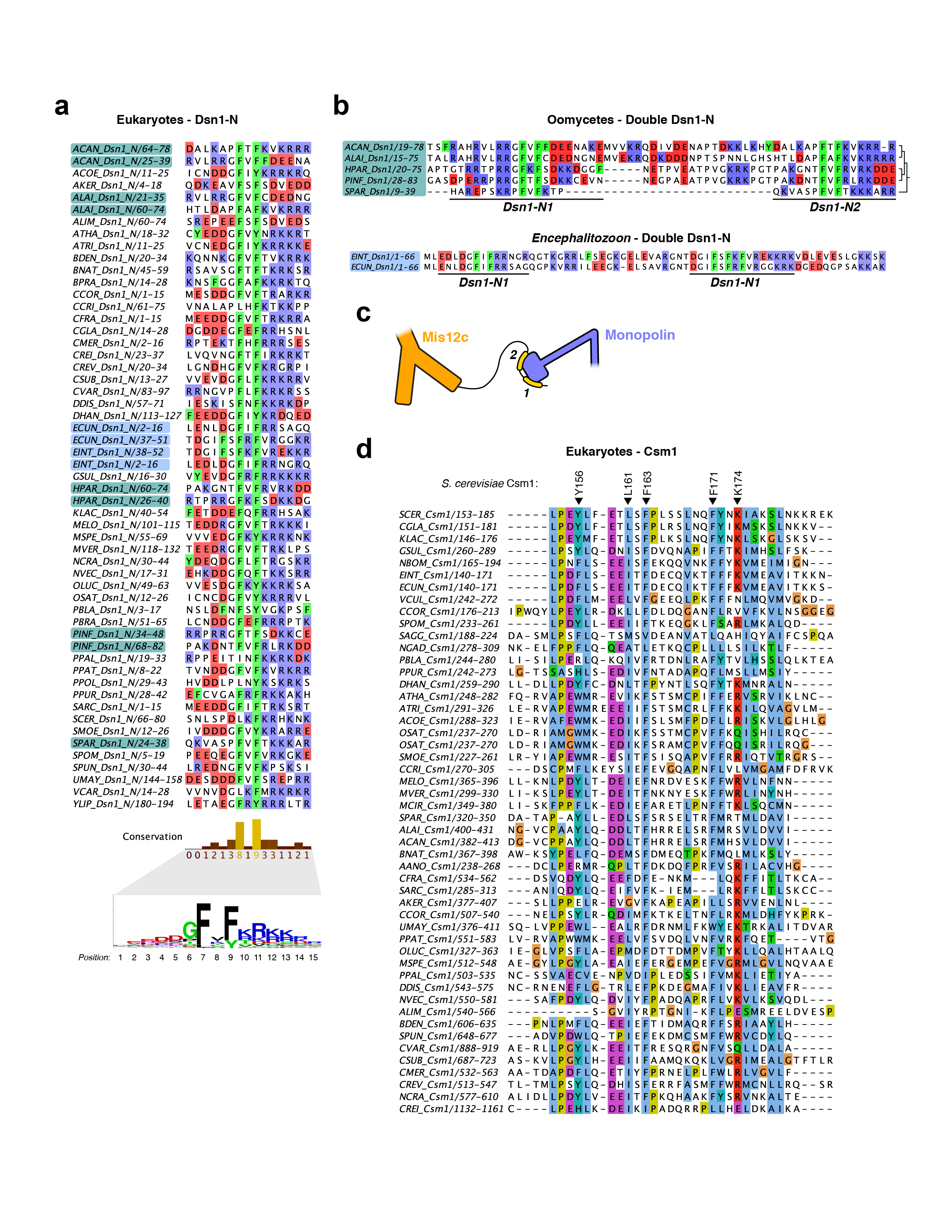
Fig. S1 Dsn1-N motif in eukaryotes**

**a** Multiple sequence alignment of Dsn1-N motifs in a set of 85 Dsn1 orthologs used in this study. Phenylalanine/tyrosine is colored green, aspartate/glutamate red, and lysine/arginine blue. Highlighted are species whose Dsn1 proteins appear to contain two Dsn1-N motifs. The four-letter abbreviations indicate the various species, for which the full names can be found in Table S1; **b** Sequence alignments of oomycete (top) and *Encephalitozoon* (bottom) Dsn1 N-terminal regions, showing the two Dsn1-N motifs; **c** Schematic of Oomycete/*Encephalitozoon* Dsn1 interacting with both protomers of a Csm1 dimer; **d** Sequence alignment of selected eukaryotic Csm1 C-terminal globular domain, with arrows indicating residues comprising the *S. cerevisiae* (SCER) Csm1 conserved hydrophobic cavity.

**
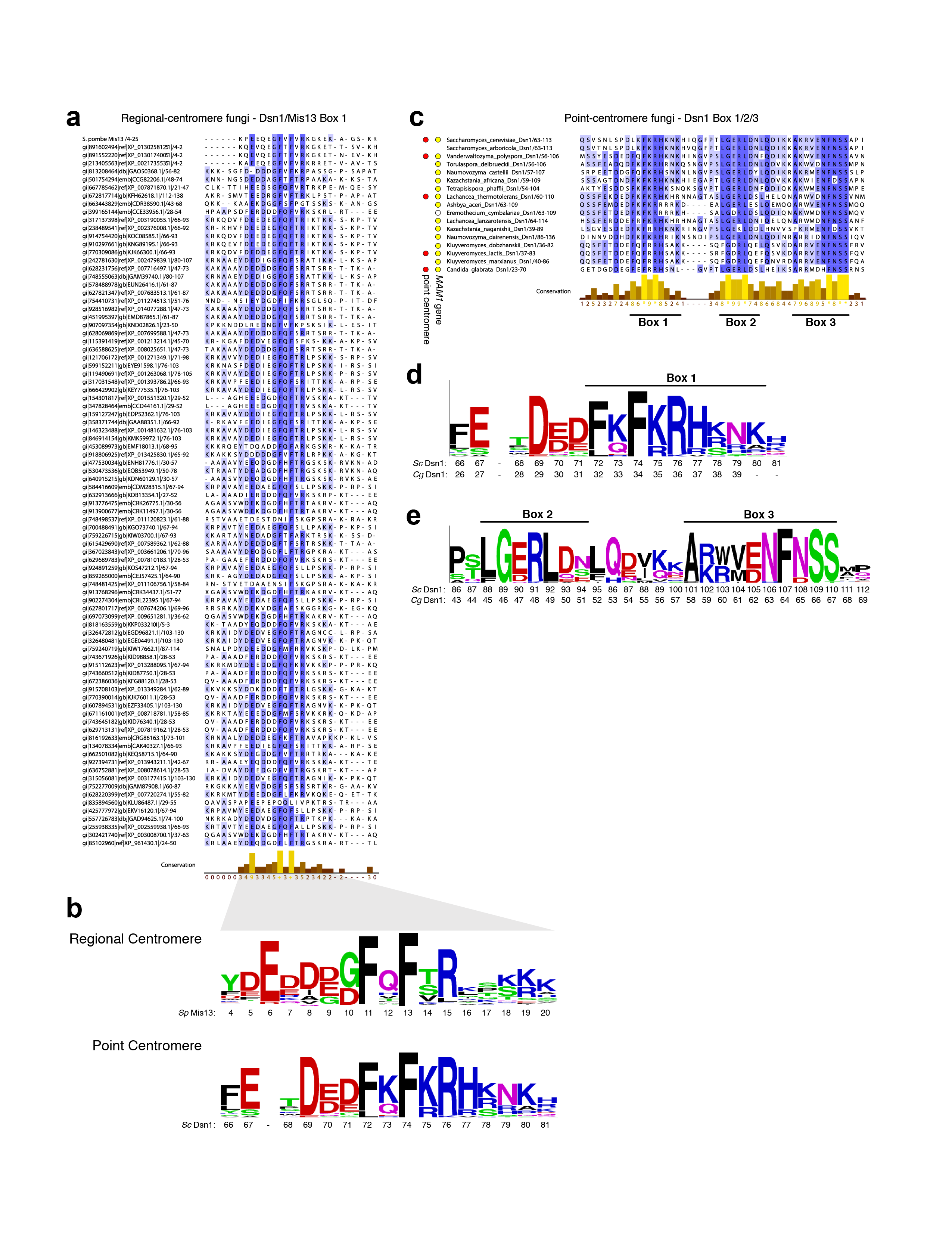
Fig. S2 Conservation of Dsn1 Boxes 1, 2, and 3**

**a** Sequence alignment of the N-terminal region of Dsn1 from 85 fungi that likely contain regional centromeres and do not possess *MAM1* orthologs; **b** *Top:* Sequence logo (Crooks et al. 2004) generated from the alignment in panel a. *Bottom:* Sequence logo for the point-centromere Dsn1 Box 1 region, from panel d, for comparison; **c** Sequence alignment of the Box 1-2-3 region of representative point-centromere fungi. Red dots on left indicate that point-centromeres have been positively identified in this organism (Meraldi et al. 2006; Gordon et al. 2011), and yellow dots indicate that the organism contains a *MAM1* ortholog (the *E. cymbalariae* contains an unannotated gene with homology to *MAM1*); **d** Sequence logo for the Dsn1 Box 1 region, generated from the alignment in panel c; **e** Sequence logo for the Dsn1 Box 2-3 region, generated from the alignment in panel c.

**
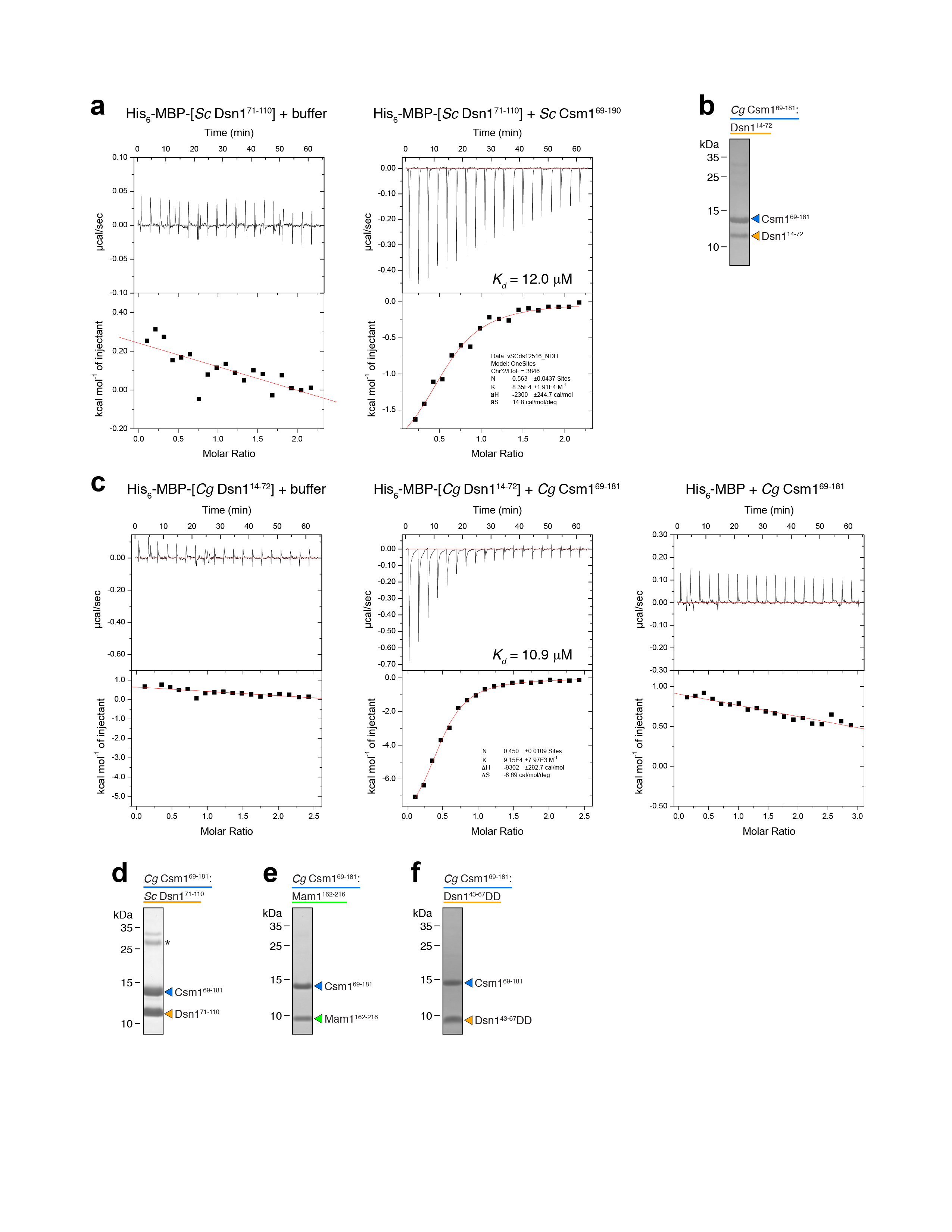
Fig. S3 Purification and biochemical analysis of Csm1-Dsn1 complexes**

**a** Isothermal titration calorimetry analysis of His_6_-MBP tagged *Sc* Dsn1^71-110^ binding *Sc* Csm1^69-190^ (*K_d_* = 12.0 μM); **b** SDS-PAGE gel of purified *Cg* Csm1^69-181^:*Cg* Dsn1^14-72^ complex; **c** Isothermal titration calorimetry analysis of His_6_-MBP tagged *Cg* Dsn1^14-72^ binding *Cg* Csm1^69-181^ (*K_d_* = 10.9 μM). *Far right:* control titration of *Cg* Csm1^69-181^ against His_6_-MBP alone. **d** SDS-PAGE gel of the *Cg* Csm1^69-181^:*Sc* Dsn1^71-110^ complex. Asterisk indicates a contaminant band. **e** SDS-PAGE gel of purified *Cg* Csm1^69-181^:*Cg* Mam1^162-216^ complex. **f** SDS-PAGE gel of purified *Cg* Csm1^69-181^:*Cg* Dsn1^43-67^DD complex

**
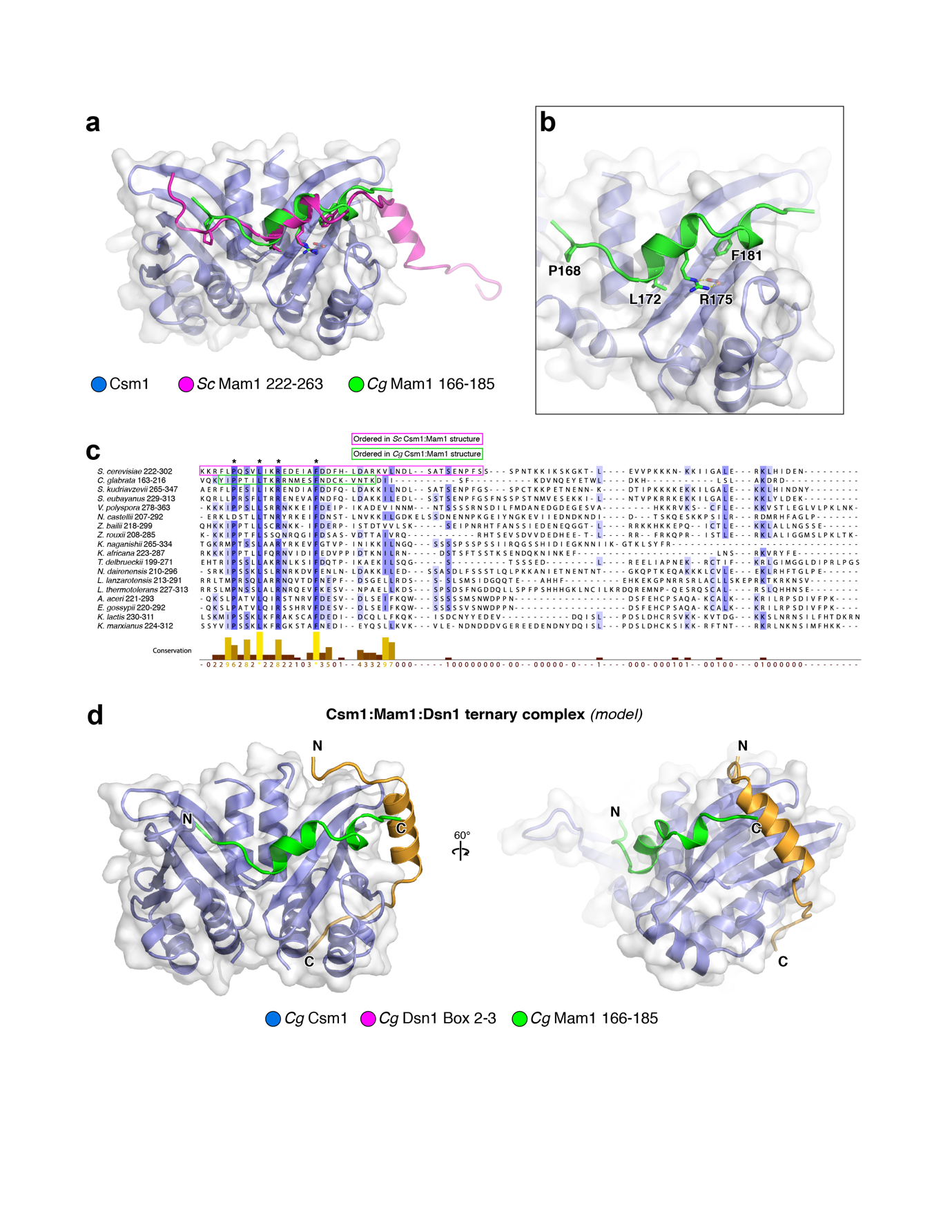
Fig. S4. Structure of a *Cg* Csm1^69-181^:*Cg* Mam1^162-216^ complex**

**a** View of the *Cg* Csm1^69-181^:*Cg* Mam1^162-216^ complex, with Csm1 shown in blue with white surface, and Dsn1 shown in green. Shown in magenta is the Mam1 chain from a prior structure of *Sc* Csm1^1-190^:*Sc* Mam1^221-302^ complex (PDB ID 5KTB) (Corbett and Harrison 2012). Shown in sticks are four highly-conserved residues in Mam1 highlighted with asterisks in panel c below; **b** Closeup view of the interaction between *Cg* Csm1and *Cg* Mam1, with highly-conserved Mam1 residues labeled; **c** Sequence alignment of budding-yeast Mam1 C-terminal regions, highlighting the limited homology in this region and showing the ordered regions of *Sc* Mam1 (magenta) and *Cg* Mam1 (green) in their respective structures; **d** Structural model of a Csm1:Mam1:Dsn1 ternary complex, assembled by overlaying the structures of *Cg* Csm1^69-181^:*Cg* Mam1^162-216^ and *Cg* Csm1^69-181^:*Cg* Dsn1^14-72^ (Box 2-3 only). The C-terminus of Mam1 is positioned close to the Dsn1 Box 2 α-helix.

**
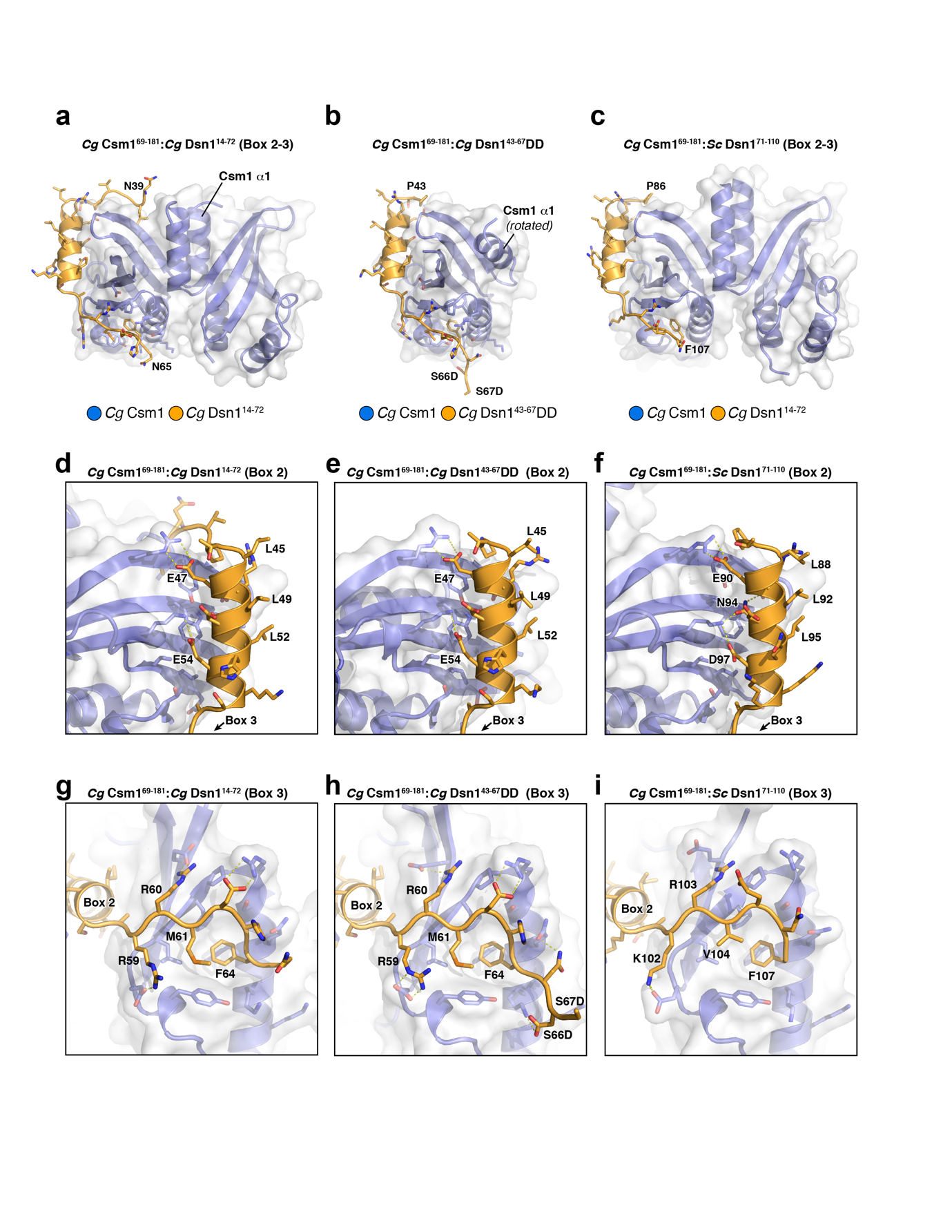
Fig. S5 Three structures show consistent Csm1-Dsn1 Box 2-3 interactions**

**a** Overall structure of the *Cg* Csm1^69-181^:*Cg* Dsn1^14-72^ complex, showing the Dsn1 Box 2-3 region; **b** Overall structure of the *Cg* Csm1^69-181^:*Cg* Dsn1^43-67^DD complex. One Csm1 protomer is shown, because of the non-canonical Csm1 dimer architecture in this crystal form (see Fig. S5c); **c** Overall structure of the *Cg* Csm1^69-181^:*Sc* Dsn1^71-110^ complex, showing the Dsn1 Box 2-3 region; **d-f** Closeup views of Dsn1 Box 2 interacting with Csm1 in *Cg* Csm1^69-181^:*Cg* Dsn1^14-72^, **d;** *Cg* Csm1^69-181^:*Cg* Dsn1^43-67^DD, **e;** and *Cg* Csm1^69-181^:*Sc* Dsn1^71-110^, **f;** **g-i** Closeup views of Dsn1 Box 3 interacting with Csm1 in *Cg* Csm1^69-181^:*Cg* Dsn1^14-72^, **g**; *Cg* Csm1^69-181^:*Cg* Dsn1^43-67^DD, **h**; and *Cg* Csm1^69-181^:*Sc* Dsn1^71-110^, **i**

**
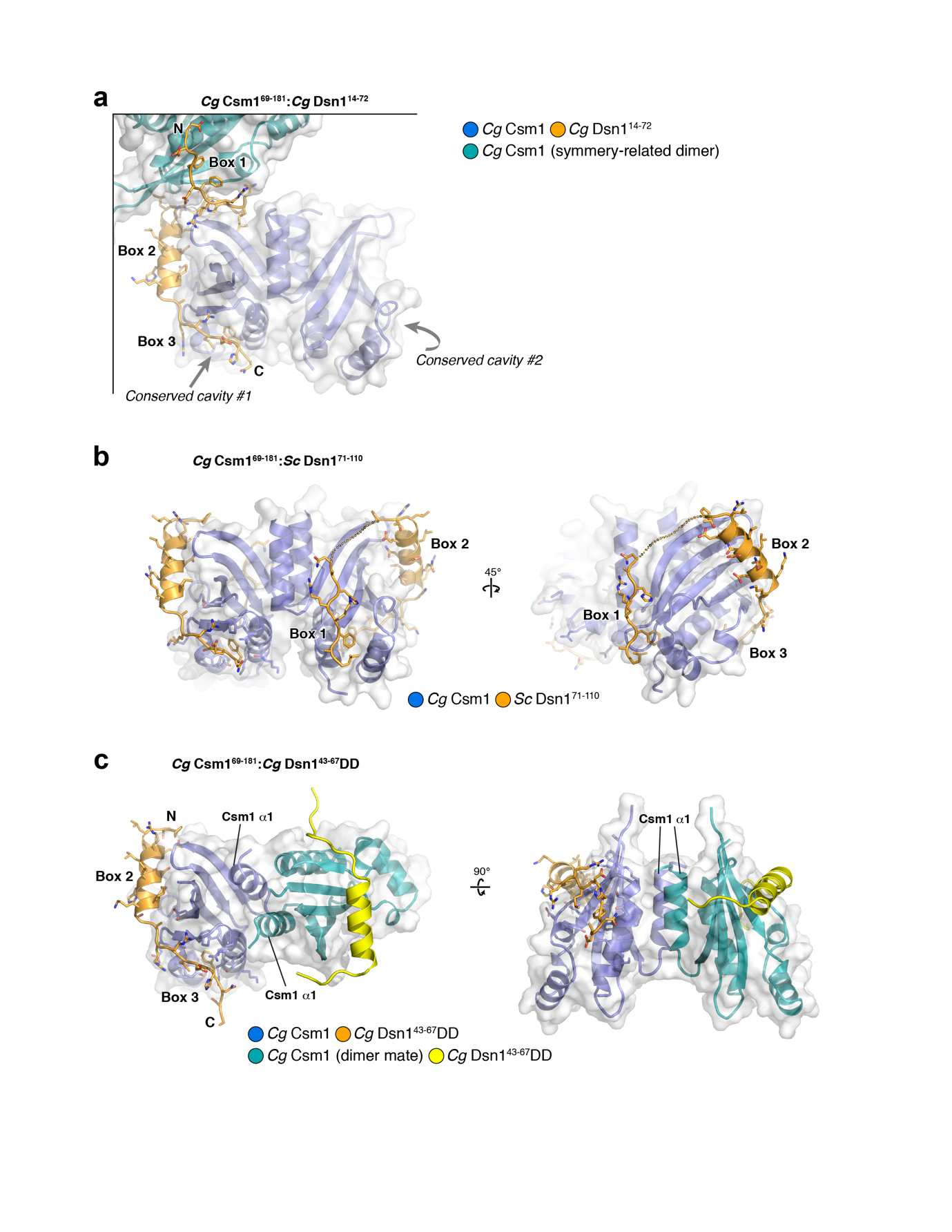
Fig. S6 Crystal packing interactions in three structures of Csm1-Dsn1 complexes**

**a** Crystal packing of the *Cg* Csm1^69-181^:*Cg* Dsn1^14-72^ complex, showing the interaction of a single Dsn1^14-72^ chain (orange with side-chains shown) with two crystallographic symmetry-related Csm1 dimers. The Dsn1 Box 2-3 region interacts with one Csm1 dimer (blue with white surface), while the Box 1 region interacts with a second crystallographic symmetry-related dimer (teal with white surface). Gray arrows indicate the locations of the two conserved hydrophobic cavities on the primary Csm1 dimer (blue). In the structure, Dsn1 Box 3 is bound to cavity #1, and cavity #2 on the dimer-related Csm1 protomer is too distant for Box 1 of the same Dsn1 protomer to simultaneously bind (this surface is occupied by Box 1 of a symmetry-related Dsn1 protomer). This crystal packing results in a 2:1 stoichiometry of Csm1 to Dsn1 in these crystals; **b** Crystal packing of the *Cg* Csm1^69-181^:*Sc* Dsn1^71-110^ complex. The asymmetric unit contains two structurally-equivalent 2:2 complexes (one shown). Each *Cg* Csm1 dimer (blue) binds two copies of *Sc* Dsn1^71-110^ (orange). In these crystals, the Dsn1 Box 1 region (residues 72-79) is ordered, and packs against a different surface of Csm1 than observed in the *Cg* Csm1^69-181^:*Cg* Dsn1^14-72^ complex. As this Csm1 surface is not well-conserved, and Dsn1 Box 1 is not tightly packed against Csm1 in this interaction (not shown), we interpret this position of Box 1 as a result of crystal packing, and not indicative of its native conformation; **b** Crystal packing of the *Cg* Csm1^69-181^:*Cg* Dsn1^43-67^DD complex. The asymmetric unit contains one Csm1 chain (blue with white surface) and one Dsn1 chain (orange with side-chains shown). The N-terminal α-helix of Csm1 is rotated ~90° from its position in all other observed Csm1 structures. A 2:2 heterotetrameric complex can be generated by crystallographic symmetry, with two Csm1 protomers interacting through their N-terminal α-helices. The unique Csm1 dimer formed by this interaction is likely the result of the low pH of the crystallization condition (pH 4.5).

**
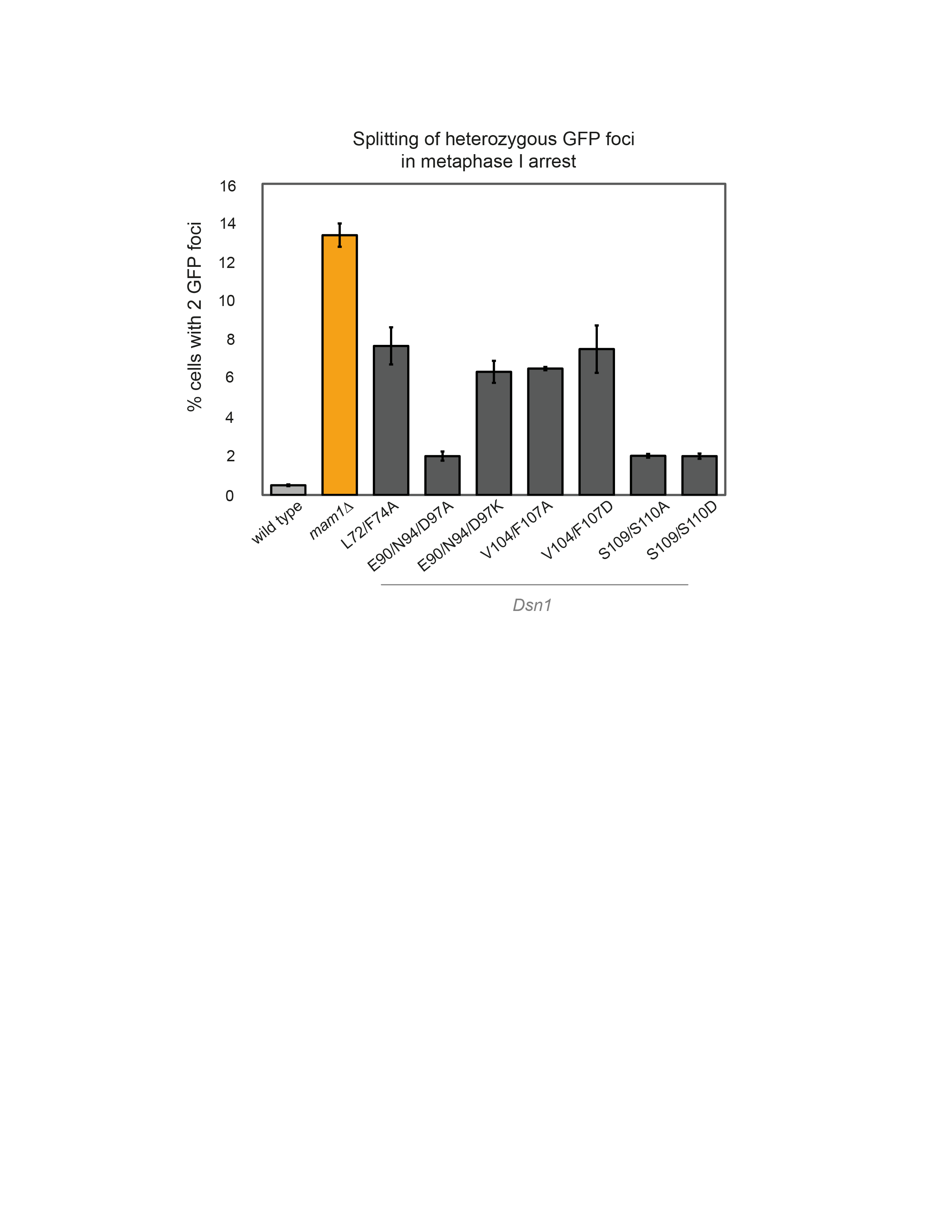
Fig. S7 Defective sister kinetochore monoorientation during meiosis I when Dsn1 N terminus is mutated**

Cells carrying *pCLB2-CDC20* and heterozygous *CEN5-GFP* were arrested in metaphase I (by depletion of Cdc20) and fixed. Cells with 2 GFP foci were scored. Shown is the average of 3 independent experiments with the exception of wild type and *mam1∆* which have 8 repeats. For each repeat, 100-200 cells were counted per repeat. Error bars are standard error of two foci proportion. Strains used are wild type (AMy20550), *mam1Δ *(AMy20551), *DSN1-L72A F74A* (AMy17415), DSN1- E90A N94A D97A (AMy22928), DSN1- E90K N94K D97K (AMy22928), *DSN1-V104A F107A (AMy24687), DSN1-V104D F107D (AMy27648), DSN1-S109A S110A* (AMy27432) and *DSN1-S109D S110D* (AMy27647)

**
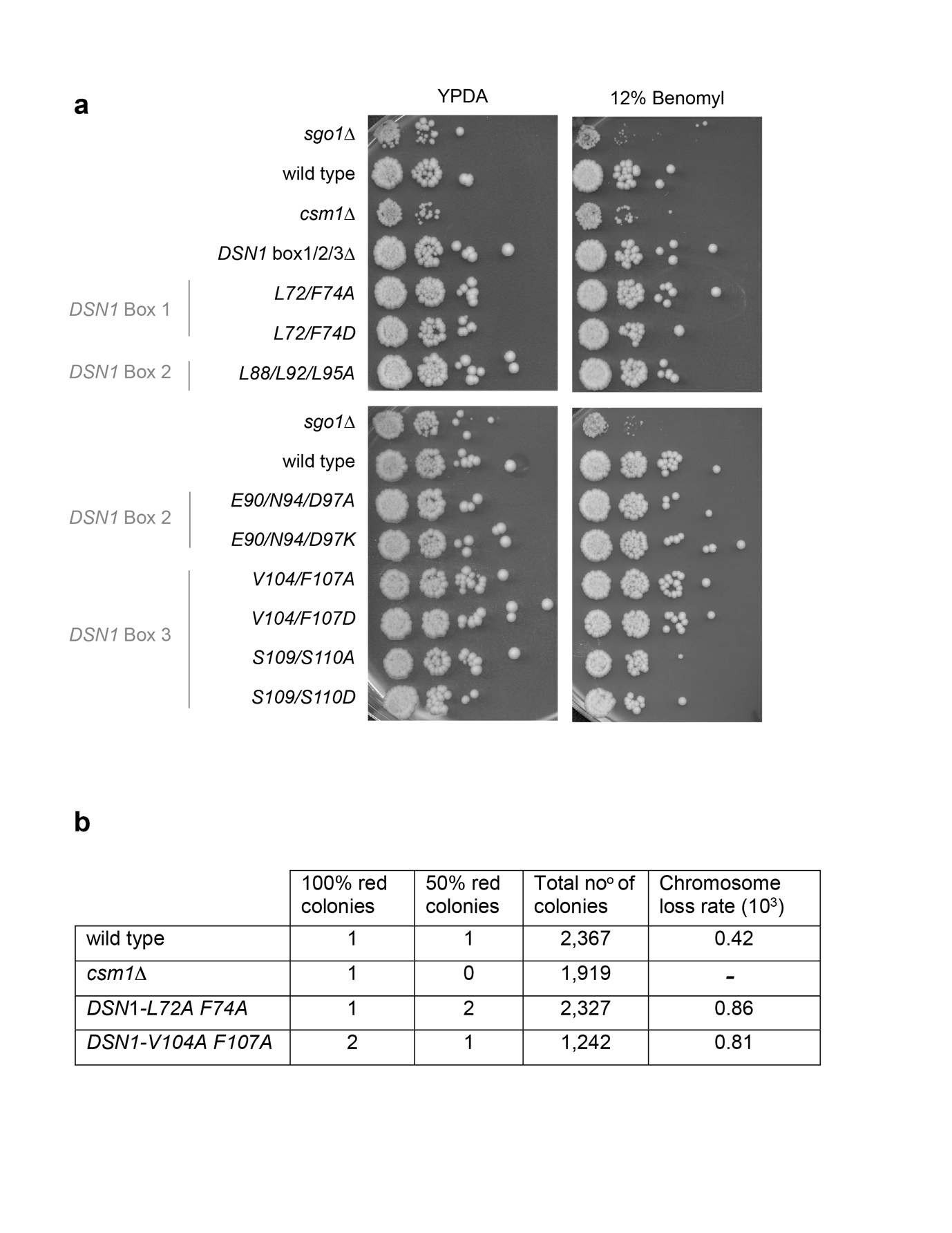
Fig. S8 Mutations in Dsn1 N terminal region do not detectably affect chromosome stability in vegetative cells**

**a** Mutations in the N-terminus of Dsn1 do not result in increased sensitivity to the microtubule-depolymerizing drug, benomyl. Cells grown in liquid media overnight, were sequentially diluted 10-fold, before spotting onto YPD or YPD with12% benomyl and incubated at 25^o^C for 3 days. Strains are AMy808 (*sgo1∆*), AMy1827 (wild type), AMy13914 (*csm1*∆), AMy 17230 *(∆110-DSN1*), AMy17222 (*DSN1-L72A F74A*), AMy17313 (*DSN1-L72D F74D*), AMy22719 (*DSN1-L88A L92A L95A*), AMy23151 (*DSN1-E90A N94A D97A*), AMy24632 (*DSN1-E90KN94KD97K*), AMy24652 (*DSN1-V104A F107A*), AMy24755 (*DSN1-V104D F107D*), AMy26803 (*DSN1-S109A S110A*), AMy24744 (*DSN1-S109D S110D*). **b** Mutations in Dsn1 Box 1 or Box 3 do not increase loss rates of an artificial minichromosome. Cells carry a non-essential minichromosome with selection marker and *SUP11*. Cells were grown in selective media to maintain the minichromosome before plating onto solid media without selection and lacking adenine. Loss of the minichromosome results in a colour change from white to red. A red/white half-sectored colony indicates loss of the chromosome fragment at the first division after plating. Loss rate is calculated as: 50% red colonies / (total colonies – 100% red colonies). Three separate *csm1∆* isolates were tested. AMy2522 and AMy2523 (wildtype), AMy27773 (*DSN1-L72A F74A*), AMy27774 (*DSN1-V104A F107A*), AMy27775, 27776 and 27777 (*csm1∆).*

**
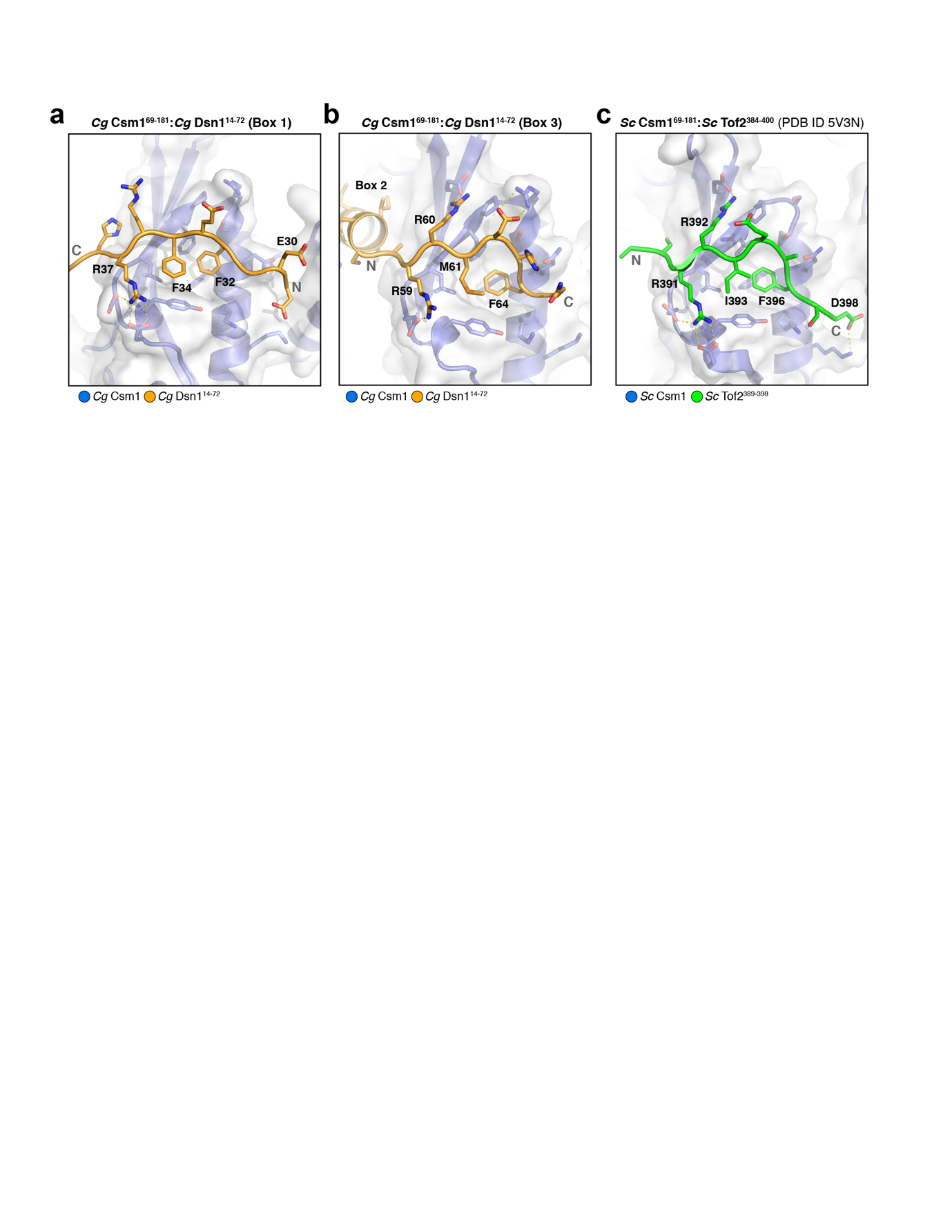
Fig. S9 Interactions of the Csm1 conserved hydrophobic cavity with three partners.**

**a** Closeup view of Dsn1 Box 1 from the structure of *Cg* Csm1^69-181^:*Cg* Dsn1^14-72^, with Csm1 in blue and Dsn1 in orange; **b** Closeup view of Dsn1 Box 3 from the structure of *Cg* Csm1^69-181^:*Cg* Dsn1^14-72^, with Csm1 in blue and Dsn1 in orange; **c** Closeup view of Tof2 from the structure of Sc Csm1^69-181^:Tof2^384-400^ (PDB ID 5V3N; (Liang et al. 2017), with Csm1 in blue and Tof2 in green. Tof2 binds Csm1 equivalently to Dsn1 Box 3, while Dsn1 Box 1 is oriented in the opposite direction (N- and C-termini of each Csm1-binding motif are labeled in gray)

**
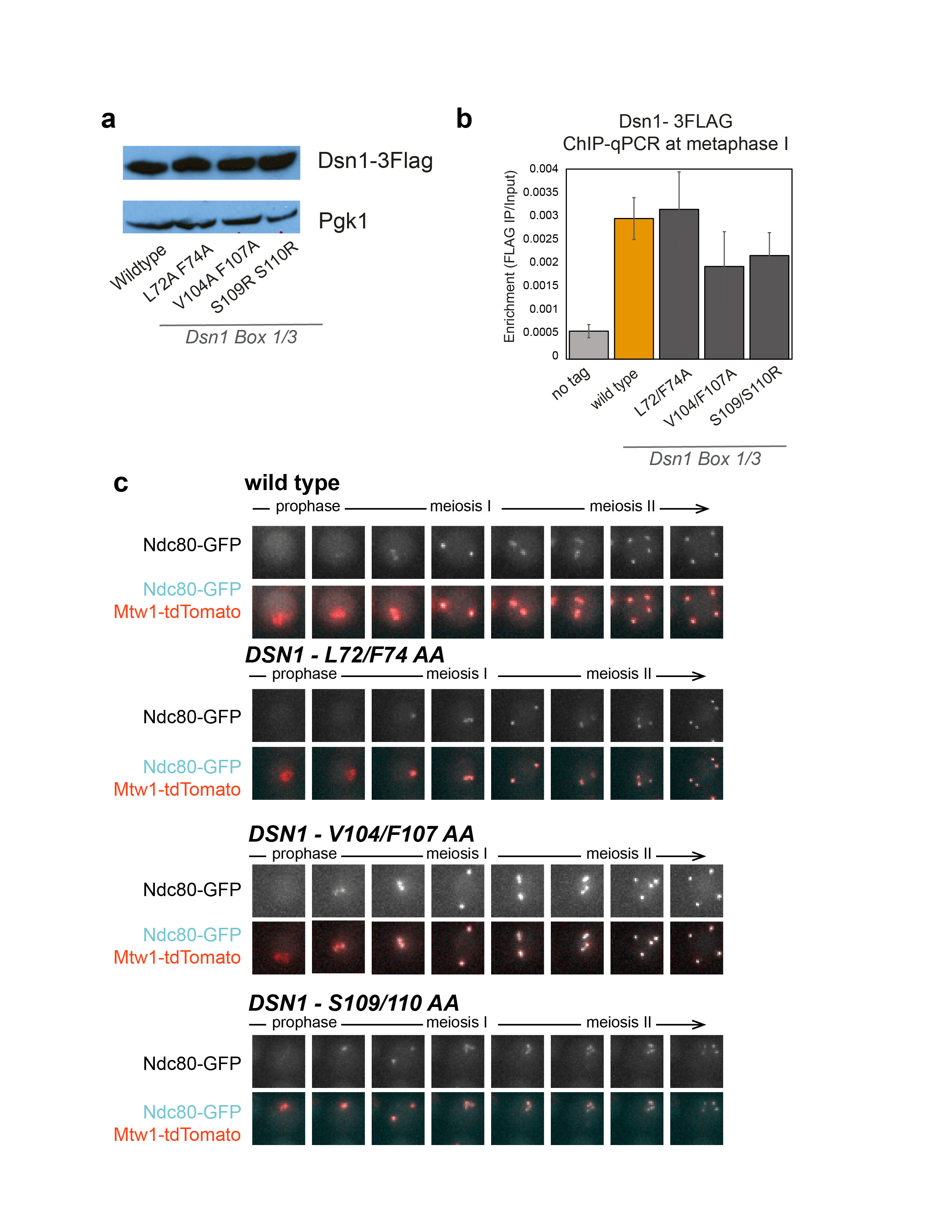
Fig. S10 Kinetochore assembly is not affected by mutations in Dsn1’s N terminus**

**a** Dsn1 mutants are expressed at wild type levels in cycling cells. Strains were as described in b and samples were isolated from exponentially growing cells; **b** Analysis of Dsn1-3Flag association with a representative centromere (*CEN13*) by anti-Flag chromatin immunoprecipitation followed by qPCR (ChIP-qPCR). Wild type (AM8291), *DSN1-L72A F74A* (AMy25618), *DSN1-V104A F107A* (AMy24669) and *DSN1-S109R S110R* (AMy24633) cells carrying *DSN1-3Flag* were arrested in metaphase I of meiosis by depletion of Cdc20. Strain AMy25617 was used as a no tag control. Shown is the average of four independent experments with error bars representing standard error. **c** Representative live cell imaging of Mtw1-TdTomato to label kinetochores and Ndc80-GFP to label the outer kinetochore. Note that Ndc80-GFP is degraded in meiotic prophase and is incorporated into kinetochores only after prophase I exit (Miller et al. 2012). Images of *DSN1-L72A F74A* (AMy26457), *DSN1-V104A F107A* (AMy26547) and *DSN1-S109A S110A* (AMy26848) strains were indistinguishable from those of wild type (AMy26337), indicating that these mutations do not affect the reassembly of the outer kinetochore subunit Ndc80 after prophase exit. Images were taken at 15 min intervals

**
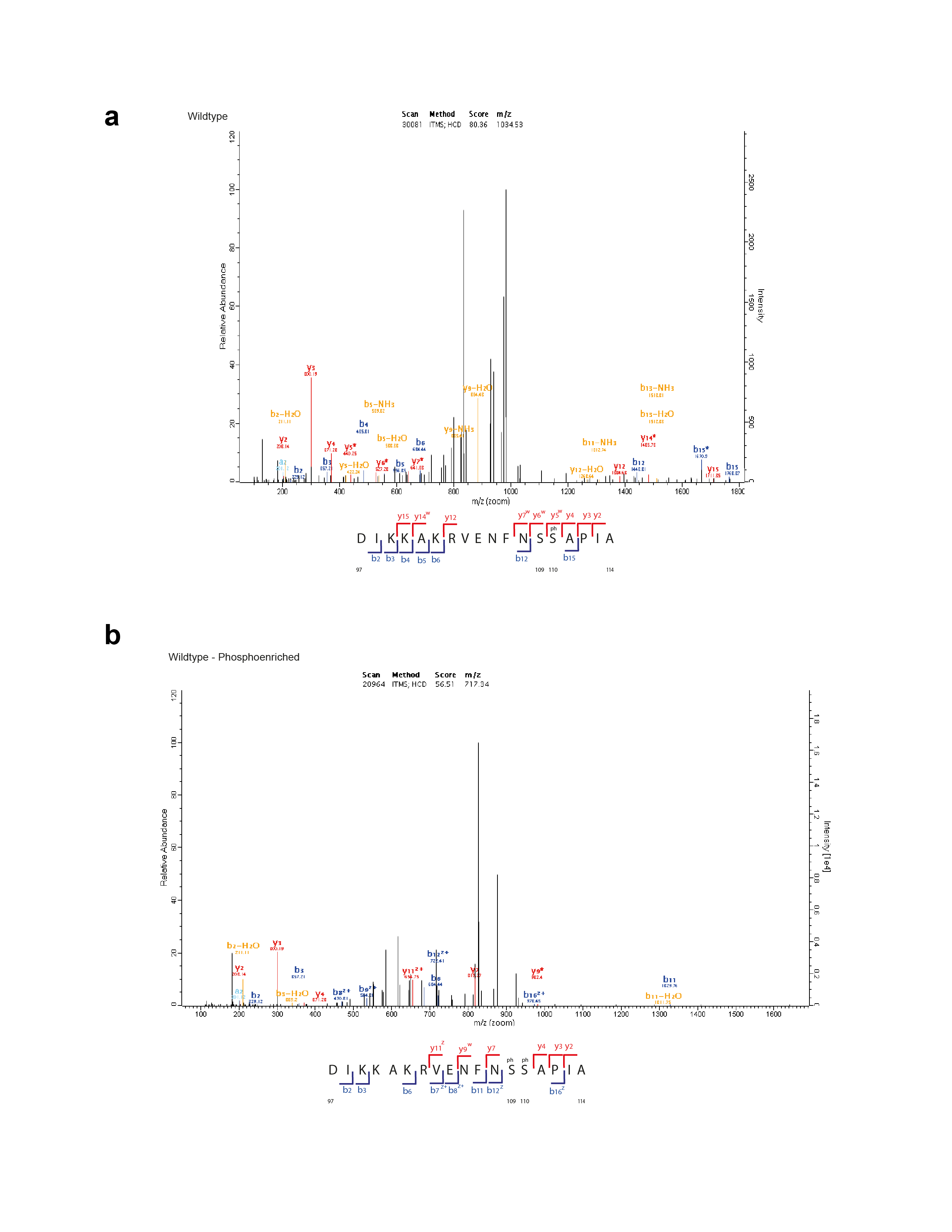
Fig. S11. Detection of phosphorylation on Dsn1 at residues S109 and S110 *in vivo* by mass spectrometry.**

Cells carrying *DSN1-6HIS-3FLAG* and *pCLB2-CDC20* Strain (AMy8291) were induced to sporulate and arrested in metaphase I by Cdc20 depletion before harvesting. Dsn1-6His-3FLAG was immunoprecipitated and analysed by mass spectrometry as described in materials and methods. Annotated MS/MS spectra of peptide Dsn1 97-114, showing detected fragments used for identification. **a** Peptide identified in sample unenriched for phosphorylation show S110 as phosphorylated. **b** Peptide identified in sample enriched for phosphorylated peptides using TiO2 spin column shows phosphorylation at both S109 S110.

**Supplementary movies**

**Movie S1 Representative movie of Box 2 mutant *DSN1- L88A L92A L95A*.**

Cells (AMy25763) carry heterozygous *CEN5-*GFP (cyan) and Mtw1-tdTomato (red) to label kinetochores, which are used to indicate progression though meiosis. Cell (A) scored as co-segregating *CEN5-GFP* to the same pole in anaphase I. Cell (B) categorized as segregating *CEN5-*GFP to opposite poles in anaphase I (sister chromatid biorientation). Full scoring of this experiment is shown in Fig. 2d.

**Movie S2 Representative movie of Box 2 mutant Dsn1- E90A N94A D97A.**

Cells (AMy25750) carry heterozygous *CEN5-*GFP (cyan) and Mtw1-tdTomato (red) to label kinetochores, which are used to indicate progression though meiosis. Cells (A and B) scored as co-segregating *CEN5-GFP* to the same pole in anaphase I. Full scoring of this experiment is shown in Fig. 2d.

**Movie S3 Representative movie of Box 2 mutant Dsn1- E90K N94K D97K.**

Cells (AMy25881) carry *CEN5-*GFP (cyan) and Mtw1-tdTomato (red) to label kinetochores, which are used to indicate progression though meiosis. Cells (A and B) scored as co-segregating *CEN5-GFP* to the same pole in anaphase I. Full scoring of this experiment is shown in Fig. 2d.

**Movie S4 Representative movie of Box 3 mutant Dsn1 V104A F107A.**

Cells (AMy25762) carry heterozygous *CEN5-*GFP (cyan) and Mtw1-tdTomato (red) to label kinetochores, which are used to indicate progression though meiosis. Cell (A) categorized as segregating *CEN5-*GFP to opposite poles in anaphase I (sister chromatid biorientation). Cells (B) scored as co-segregating *CEN5-GFP* to the same pole in anaphase I. Full scoring of this experiment is shown in Fig. 3d.

**Movie S5 Representative movie of Box 3 mutant Dsn1 V104D F107D.**

Cells (AMy26475) carry heterozygous *CEN5-*GFP (cyan) and Mtw1-tdTomato (red) to label kinetochores, which are used to indicate progression though meiosis. Cells (A and B) categorized as segregating *CEN5-*GFP to opposite poles in anaphase I (sister chromatid biorientation). Full scoring of this experiment is shown in Fig. 3d.

**Movie S6 Representative movie of Box 2 mutant Dsn1- S109D S110D.**

Cells (AMy27009) carry heterozygous *CEN5-*GFP (cyan) and Mtw1-tdTomato (red) to label kinetochores, which are used to indicate progression though meiosis. Cells (A, B and C) scored as co-segregating *CEN5-GFP* to the same pole in anaphase I. Full scoring of this experiment is shown in Fig. 3d.

**Movie S7 Representative movie of Box 3 mutant Dsn1 S109A S110A.**

Cells carry heterozygous *CEN5-*GFP (cyan) and Mtw1-tdTomato (red) to label kinetochores, which are used to indicate progression though meiosis. Cell (A) categorized as segregating *CEN5-*GFP to opposite poles in anaphase I (sister chromatid biorientation). Cell (B) was not scored as two distinct attempts at division cannot be identified so it is unclear which stage of meiosis the cell is in and therefore at what stage sister chromatids first divide. Cells (C) scored as co-segregating *CEN5-GFP* to the same pole in anaphase I. Full scoring of this experiment appears in Fig. 3d.

**Movie S8 Representative movie of Box 1 mutant *DSN1-* *L72A F74A*.**

Cells (AMy25821) carry heterozygous *CEN5-*GFP (cyan) and Mtw1-tdTomato (red) to label kinetochores, which are used to indicate progression though meiosis. Cell (A) categorized as segregating *CEN5-*GFP to opposite poles in anaphase I (sister chromatid biorientation). Cell (B) was not scored as it already progressed too far in meiosis before imaging began. Cell (C) categorized as segregating *CEN5-*GFP to opposite poles in anaphase I (sister chromatid biorientation). Although this cell attempts 2 divisions, the second division fails to correctly segregate the kinetochore marker into 4 separate foci. Cells (D) scored as co-segregating *CEN5-GFP* to the same pole in anaphase I. Full scoring of this experiment is shown in Fig. 4c.

**Movie S9 Representative movie of Box1 mutant *DSN1- L72D F74D*.**

Cells (AMy26543) carry heterozygous *CEN5-*GFP (cyan) and Mtw1-tdTomato (red) to label kinetochores, which are used to indicate progression though meiosis. Cell (A)categorized as segregating *CEN5-*GFP to opposite poles in anaphase I (sister chromatid biorientation). Cells (B and C) scored as co-segregating *CEN5-GFP* to the same pole in anaphase I. Full scoring of this experiment is shown in Fig. 4c.

**Table S1. Phylogenetic profiles of kinetochore proteins**

See supplementary table 1 .xlsx file. The first tab (‘Presence-absence’) shows the presence (1) and absences (0) of kinetochore proteins in a set of 109 proteomes, representative of the diversity of throughout the eukaryotic tree of life. Tab2 contains all the pairwise pearson correlation scores for the phylogenetic profile of the kinetochore proteins in this study. Tab3 (‘co-evolution’) shows an overview similar to Fig. 1c.

**Table S2.** **Yeast strains used in this study**

| **Strain number** | **Haploid/ Diploid** | **Genotype** |
| --- | --- | --- |
| AMy808 | haploid | *MATa sgo1Δ::KanMX6* |
| AMy1827 | haploid | *MATa* |
| AMy1828 | haploid | *MATalpha* |
| AMy1835 | diploid | *MATa MATalpha* |
| AMy1932 | haploid | *MATa mam1∆::TRP1* |
| AMy1947 | haploid | *MATalpha mam1∆::TRP1* |
| AMy2522 | haploid | *MATa CFIII (CEN3.L.YPH983) HIS3 SUP11* |
| AMy2523 | haploid | *MATa CFIII (CEN3.L.YPH983) HIS3 SUP11* |
| AMy8067 | haploid | *MATa cdc20::pCLB2-CDC20::KanMX6 MATalpha cdc20::pCLB2-CDC20::KanMX6* |
| AMy8291 | haploid | *MATalpha DSN1-6HIS-3FLAG::URA3 cdc20::pCLB2-3HA-CDC20::KanMX6 MATa DSN1-6HIS-3FLAG::URA3  cdc20::pCLB2-3HA-CDC20::KanMX* |
| AMy12538 | diploid | *MATa DSN1-6HIS-3FLAG::URA3 cdc20::pCLB2-CDC20::KanMX6 mam1Δ::KANMX6 MATalpha DSN1-6HIS-3FLAG::URA3 cdc20::pCLB2-CDC20::KanMX6 mam1Δ::KANMX6* |
| AMy13914 | haploid | *MATa csm1Δ::KANMX6* |
| AMy14942 | diploid | *MATalpha GAL-NDT80::TRP1 ura3::pGPD1-GAL4(848).ER::URA3 MAM1-yeGFP::KITRP1 MTW1-tdTomato::NAT MATa GAL-NDT80::TRP1 ura3::pGPD1-GAL4(848).ER::URA3 MAM1-yeGFP::KITRP1 MTW1-tdTomato::NAT* |
| AMy15096 | diploid | *MATalpha csm1::KANMX6 GAL-NDT80::TRP1 ura3::pGPD1-GAL4(848).ER::URA3 MAM1-yeGFP::KITRP1 MTW1-tdTomato::NAT MATa, csm1::KANMX6 GAL-NDT80::TRP1 ura3::pGPD1-GAL4(848).ER::URA3 MAM1-yeGFP::KITRP1 MTW1-tdTomato::NAT* |
| AMy17120 | haploid | *MATalpha dsn1::DSN1(L72A F74A)-6HIS-3FLAG::URA3 cdc20::pCLB2-3HA-CDC20::KanMX6 promURA3::TetR::GFP::LEU2 tetOx224-HIS3* |
| AMy17123 | haploid | *MATalpha dsn1::DSN1(L72D F74D)-6HIS-3FLAG::URA3*  *cdc20::pCLB2-3HA-CDC20::KanMX6 promURA3::TetR::GFP::LEU2 tetOx224-HIS3* |
| AMy17222 | haploid | *MATalpha dsn1::DSN1(L72A F74A)-6HIS-3FLAG::URA3* |
| AMy17223 | haploid | *MATa dsn1::DSN1(L72A F74A)-6HIS-3FLAG::URA3* |
| AMy17230 | haploid | *MATa KANMX6-Δ110DSN1-6HIS-3FLAG::URA3* |
| AMy17232 | haploid | *MATalpha KANMX6-Δ78DSN1-6HIS-3FLAG::URA3* |
| AMy17313 | haploid | *MATalpha dsn1::DSN1(L72D F74D)-6HIS-3FLAG::URA3* |
| AMy17373 | haploid | *MATa dsn1::DSN1(L72D F74D)-6HIS-3FLAG::URA3* |
| AMy17415 | diploid | *MATalpha dsn1::DSN1(L72A F74A)-6HIS-3FLAG::URA3 cdc20::pCLB2-3HA-CDC20::KanMX6 promURA3::TetR::GFP::LEU2 tetOx224-HIS3 (Tomo’s centromeric) MATa dsn1::DSN1(L72A F74A)-6HIS-3FLAG::URA3 cdc20::pCLB2-3HA-CDC20::KanMX6* |
| AMy17505 | haploid | *MATalpha KANMX6-Δ110DSN1-6HIS-3FLAG::URA3* |
| AMy17507 | haploid | *MATa KANMX6-Δ110DSN1-6HIS-3FLAG::URA3* |
| AMy20550 | diploid | *MATa cdc20::pCLB2-3HA-CDC20::KanMX6 MATalpha cdc20::pCLB2-3HA-CDC20::KanMX6 promURA3::TetR::GFP::LEU2 tetOx224-HIS3 (Tomo’s centromeric)* |
| AMy20551 | diploid | *MATa cdc20::pCLB2-3HA-CDC20::KanMX6 mam1::TRP1 MATalpha cdc20::pCLB2-3HA-CDC20::KanMX6 promURA3::TetR::GFP::LEU2 tetOx224-HIS3 (Tomo’s centromeric) mam1::TRP1* |
| AMy21920 | haploid | *MATa dsn1::DSN1(E90A N94A D97A)-6HIS-3FLAG::URA3* |
| AMy21921 | haploid | *MATa dsn1::DSN1(L88A L92A L95A)-6HIS-3FLAG::URA3* |
| AMy22719 | haploid | *MATalpha dsn1::DSN1(L88A L92A L95A)-6HIS-3FLAG::URA3* |
| AMy22794 | diploid | *MATa dsn1::DSN1(L88A L92A L95A)-6HIS-3FLAG::URA3 cdc20::pCLB2-3HA-CDC20::KanMX6 MATalpha dsn1::DSN1(L88A L92A L95A)-6HIS-3FLAG::URA3 cdc20::pCLB2-3HA-CDC20::KanMX6 promURA3::TetR::GFP::LEU2, tetOx224-HIS3 (Tomo’s centromeric)* |
| AMy22928 | diploid | *MATalpha dsn1::DSN1(E90A N94A D97A)-6HIS-3FLAG::URA3 cdc20::pCLB2-3HA-CDC20::KanMX6 MATa dsn1::DSN1(E90A N94A D97A)-6HIS-3FLAG::URA3 cdc20::pCLB2-3HA-CDC20::KanMX6 promURA3::TetR::GFP::LEU2 tetOx224-HIS3 (Tomo’s centromeric)* |
| AMy23151 | haploid | *MATalpha dsn1::DSN1(E90A N94A D97A)-6HIS-3FLAG::URA3* |
| AMy23152 | haploid | *MATa dsn1::DSN1(E90A N94A D97A)-6HIS-3FLAG::URA3* |
| AMy23695 | diploid | *MATalpha cdc20::pCLB2-3HA-CDC20::KanMX6 NDC10-6HA::HIS3MX6 MAM1-9MYC::TRP1 MATa cdc20::pCLB2-3HA-CDC20::KanMX6 NDC10-6HA::HIS3MX6 MAM1-9MYC::TRP1* |
| AMy24624 | haploid | *MATa dsn1::DSN1(V104A F107A)-6HIS-3FLAG::URA3* |
| AMy24629 | haploid | *MATa dsn1::DSN1(E90K N94K D97K)-6HIS-3FLAG::URA3* |
| AMy24632 | haploid | *MATalpha dsn1::DSN1(E90K N94K D97K)-6HIS-3FLAG::URA3* |
| AMy24633 | diploid | *MATa dsn1::DSN1(S109R S110R)-6HIS-3FLAG::URA3 MAM1-9MYC::TRP1*  *cdc20::pCLB2-3HA-CDC20::KanMX6 MATalpha dsn1::DSN1(S109R S110R)-6HIS-3FLAG::URA3 MAM1-9MYC::TRP1 cdc20::pCLB2-3HA-CDC20::KanMX6* |
| AMy24652 | haploid | *MATalpha dsn1::DSN1(V104A F107A)-6HIS-3FLAG::URA3* |
| AMy24669 | diploid | *MATa dsn1::DSN1(V104A F107A)-6HIS-3FLAG::URA3 MAM1-9MYC::TRP1 cdc20::pCLB2-3HA-CDC20::KanMX6 MATalpha dsn1::DSN1(V104A F107A)-6HIS-3FLAG::URA3 MAM1-9MYC::TRP1 cdc20::pCLB2-3HA-CDC20::KanMX6* |
| AMy24687 | diploid | *MATa dsn1::DSN1(V104A F107A)-6HIS-3FLAG::URA3 cdc20::pCLB2-3HA-CDC20::KanMX6 MATalpha dsn1::DSN1(V104A F107A)-6HIS-3FLAG::URA3 cdc20::pCLB2-3HA-CDC20::KanMX6 promURA3::TetR::GFP::LEU2 tetOx224-HIS3 (Tomo’s centromeric)* |
| AMy24688 | haploid | *MATa dsn1::DSN1(S109D S110D)-6HIS-3FLAG::URA3* |
| AMy24744 | haploid | *MATalpha dsn1::DSN1(S109D S110D)-6HIS-3FLAG::URA3* |
| AMy24750 | diploid | *MATa dsn1::DSN1(E90A N94A D97A)-6HIS-3FLAG::URA3 MAM1-9MYC::TRP1 cdc20::pCLB2-3HA-CDC20::KanMX6 MATalpha dsn1::DSN1(E90A N94A D97A)-6HIS-3FLAG::URA3 MAM1-9MYC::TRP1 cdc20::pCLB2-3HA-CDC20::KanMX6* |
| AMy24755 | haploid | *Mata dsn1::DSN1(V104D F107D)-6HIS-3FLAG::URA3* |
| AMy24857 | haploid | *MATalpha dsn1::DSN1(V104D F107D)-6HIS-3FLAG::URA3 cdc20::pCLB2-3HA-CDC20::KanMX6* |
| AMy24858 | haploid | *MATa dsn1::DSN1(V104D F107D)-6HIS-3FLAG::URA3* |
| AMy25110 | haploid | *MATalpha dsn1::DSN1(V104D F107D)-6HIS-3FLAG::URA3* |
| AMy25111 | diploid | *MATalpha dsn1::DSN1(L72D F74D)-6HIS-3FLAG::URA3 MAM1-9MYC::TRP1 cdc20::pCLB2-3HA-CDC20::KanMX6 MATa dsn1::DSN1(L72D F74D)-6HIS-3FLAG::URA3 MAM1-9MYC::TRP1 cdc20::pCLB2-3HA-CDC20::KanMX6* |
| AMy25615 | diploid | *MATa dsn1::DSN1(E90K N94K D97K)-6HIS-3FLAG::URA3 cdc20::pCLB2-3HA-CDC20::KanMX6 MATalpha dsn1::DSN1(E90K N94K D97K)-6HIS-3FLAG::URA3 cdc20::pCLB2-3HA-CDC20::KanMX6 promURA3::TetR::GFP::LEU2 tetOx224-HIS3 (Tomo’s centromeric)* |
| AMy25617 | diploid | *MATalpha MAM1-9MYC::TRP1 cdc20::pCLB2-3HA-CDC20::KanMX6 MATa MAM1-9MYC::TRP1 cdc20::pCLB2-3HA-CDC20::KanMX6* |
| AMy25618 | diploid | *MATalpha dsn1::DSN1(L72A F74A)-6HIS-3FLAG::URA3 MAM1-9MYC::TRP1 cdc20::pCLB2-3HA-CDC20::KanMX6 MATa dsn1::DSN1(L72A F74A)-6HIS-3FLAG::URA3 MAM1-9MYC::TRP1 cdc20::pCLB2-3HA-CDC20::KanMX6* |
| AMy25750 | diploid | *MATalpha dsn1::DSN1(E90A N94A D97A)-6HIS-3FLAG::URA3 PDS1-tdTomato-KITRP1 MTW1-tdTomato::NAT MATa dsn1::DSN1(E90A N94A D97A)-6HIS-3FLAG::URA3 PDS1-tdTomato-KITRP1 MTW1-tdTomato::NAT promURA3::TetR::GFP::LEU2 tetOx224-HIS3* |
| AMy25762 | diploid | *MATalpha dsn1::DSN1(V104A F107A)-6HIS-3FLAG::URA3 PDS1-tdTomato-KITRP1 MTW1-tdTomato::NAT MATa dsn1::DSN1(V104A F107A)-6HIS-3FLAG::URA3 PDS1-tdTomato-KITRP1 MTW1-tdTomato::NAT promURA3::TetR::GFP::LEU2 tetOx224-HIS3* |
| AMy25763 | diploid | *MATa dsn1::DSN1(L88A L92A L95A)-6HIS-3FLAG::URA3 MTW1-tdTomato::NAT PDS1-tdTomato-KITRP1 MATalpha dsn1::DSN1(L88A L92A L95A)-6HIS-3FLAG::URA3 PDS1-tdTomato-KITRP1 MTW1-tdTomato::NAT promURA3::TetR::GFP::LEU2 tetOx224-HIS3* |
| AMy25821 | diploid | *MATalpha dsn1::DSN1(L72A F74A)-6HIS-3FLAG::URA3 MTW1-tdTomato::NAT PDS1-tdTomato-KITRP1 MATa dsn1::DSN1(L72A F74A)-6HIS-3FLAG::URA3 PDS1-tdTomato-KITRP1 MTW1-tdTomato::NAT promURA3::TetR::GFP::LEU2 tetOx224-HIS3* |
| AMy25881 | diploid | *MATalpha dsn1::DSN1(E90K N94K D97K)-6HIS-3FLAG::URA3 PDS1-tdTomato-KITRP1 MTW1-tdTomato::NAT MATa dsn1::DSN1(E90K N94K D97K)-6HIS-3FLAG::URA3*  *PDS1-tdTomato-KITRP1 MTW1-tdTomato::NAT promURA3::TetR::GFP::LEU2 tetOx224-HIS3* |
| AMy25883 | haploid | *MATa dsn1::DSN1(L72A F74A S109A S110A)-6HIS-3FLAG::URA3* |
| AMy25932 | diploid | *MATalpha DSN1-6HIS-3FLAG::URA3 cdc20::pCLB2-3HA-CDC20::KanMX6 MATa DSN1-6HIS-3FLAG::URA3  cdc20::pCLB2-3HA-CDC20::KanMX PDS1-tdTomato-KITRP1 MTW1-tdTomato::NAT MATa DSN1-6HIS-3FLAG::URA3 PDS1-tdTomato-KITRP1 MTW1-tdTomato::NAT promURA3::TetR::GFP::LEU2 tetOx224-HIS3* |
| AMy26337 | diploid | *MATa DSN1-6HIS-3FLAG::URA3 Ndc80-yEGFP::KanMX MTW1-tdTomato::NAT MATalpha DSN1-6HIS-3FLAG::URA3 Ndc80-yEGFP::KanMX MTW1-tdTomato::NAT* |
| AMy26426 | haploid | *MATa dsn1::DSN1(S109A S110A)-6HIS-3FLAG::URA3* |
| AMy26546 | diploid | *MATalpha mam1Δ::TRP1 PDS1-tdTomato-KITRP1 MTW1-tdTomato::NAT MATa mam1Δ::TRP1 PDS1-tdTomato-KITRP1 MTW1-tdTomato::NAT promURA3::TetR::GFP::LEU2 , tetOx224-HIS3 (Tomo’s centromeric)* |
| AMy26457 | diploid | *MATa dsn1::DSN1(L72A F74A)-6HIS-3FLAG::URA3 Ndc80-yEGFP::KanMX MTW1-tdTomato::NAT MATalpha dsn1::DSN1(L72A F74A)-6HIS-3FLAG::URA3 Ndc80-yEGFP::KanMX MTW1-tdTomato::NAT* |
| AMy26475 | diploid | *MATalpha dsn1::DSN1(V104D F107D)-6HIS-3FLAG::URA3 cdc20::pCLB2-3HA-CDC20::KanMX6 PDS1-tdTomato-KITRP1 MTW1-tdTomato::NAT MATa dsn1::DSN1(V104D F107D)-6HIS-3FLAG::URA3 PDS1-tdTomato-KITRP1 MTW1-tdTomato::NAT promURA3::TetR::GFP::LEU2 tetOx224-HIS3* |
| AMy26476 | diploid | *MATa dsn1::DSN1(S109D S110D)-6HIS-3FLAG::URA3 MAM1-9MYC::TRP1 cdc20::pCLB2-3HA-CDC20::KanMX6 MATalpha dsn1::DSN1(S109D S110D)-6HIS-3FLAG::URA3 MAM1-9MYC::TRP1 cdc20::pCLB2-3HA-CDC20::KanMX6* |
| AMy26543 | diploid | *MATalpha dsn1::DSN1(L72D F74D)-6HIS-3FLAG::URA3 MTW1-tdTomato::NAT PDS1-tdTomato-KITRP1 MATa dsn1::DSN1(L72D F74D)-6HIS-3FLAG::URA3 MTW1-tdTomato::NAT PDS1-tdTomato-KITRP1 promURA3::TetR::GFP::LEU2 tetOx224-HIS3* |
| AMy26547 | diploid | *MATa dsn1::DSN1(V104A F107A)-6HIS-3FLAG::URA3 Ndc80-yEGFP::KanMX*  *MTW1-tdTomato::NAT MATalpha dsn1::DSN1(V104A F107A)-6HIS-3FLAG::URA3 Ndc80-yEGFP::KanMX MTW1-tdTomato::NAT* |
| AMy26727 | haploid | *MATa dsn1::DSN1(L72A F74A V104A F107A)-6HIS-3FLAG::URA3* |
| AMy26728 | haploid | *MATa dsn1::DSN1(L72A F74A V104A F107A S109A S110A)-6HIS-3FLAG::URA3* |
| AMy26778 | diploid | *MATa dsn1::DSN1(V104D F107D)-6HIS-3FLAG::URA3 cdc20::pCLB2-3HA-CDC20::KanMX6 MAM1-9MYC::TRP1 MATalpha dsn1::DSN1(V104D F107D)-6HIS-3FLAG::URA3 cdc20::pCLB2-3HA-CDC20::KanMX6 MAM1-9MYC::TRP1* |
| AMy26800 | diploid | *MATa dsn1::DSN1(S109A S110A)-6HIS-3FLAG::URA3 MAM1-9MYC::TRP1 cdc20::pCLB2-3HA-CDC20::KanMX6 MATalpha dsn1::DSN1(S109A S110A)-6HIS-3FLAG::URA3 MAM1-9MYC::TRP1 cdc20::pCLB2-3HA-CDC20::KanMX6* |
| AMy26803 | haploid | *MATalpha dsn1::DSN1(S109A S110A)-6HIS-3FLAG::URA3* |
| AMy26828 | diploid | *MATa dsn1::DSN1(S109A S110A)-6HIS-3FLAG::URA3 MTW1-tdTomato::NAT PDS1-tdTomato-KITRP1 MATalpha dsn1::DSN1(S109A S110A)-6HIS-3FLAG::URA3 MTW1-tdTomato::NAT PDS1-tdTomato-KITRP1 promURA3::TetR::GFP::LEU2 tetOx224-HIS3* |
| AMy26848 | diploid | *MATa dsn1::DSN1(S109A S110A)-6HIS-3FLAG::URA3 Ndc80-yEGFP::KanMX*  *MTW1-tdTomato::NAT MATalpha dsn1::DSN1(S109A S110A)-6HIS-3FLAG::URA3*  *Ndc80-yEGFP::KanMX MTW1-tdTomato::NAT* |
| AMy26947 | diploid | *MATa dsn1::DSN1(S109D S110D)-6HIS-3FLAG::URA3 GAL-NDT80::TRP1 ura3::pGPD1-GAL4(848).ER::URA3 MAM1-yeGFP::KITRP1 MTW1-tdTomato::NAT MATalpha dsn1::DSN1(S109D S110D)-6HIS-3FLAG::URA3*  *GAL-NDT80::TRP1 ura3::pGPD1-GAL4(848).ER::URA3 MAM1-yeGFP::KITRP1 MTW1-tdTomato::NAT* |
| AMy26963 | diploid | *MATalpha dsn1::DSN1(S109A S110A)-6HIS-3FLAG::URA3 GAL-NDT80::TRP1, ura3::pGPD1-GAL4(848).ER::URA3 MAM1-yeGFP::KITRP1 MTW1-tdTomato::NAT MATa dsn1::DSN1(S109A S110A)-6HIS-3FLAG::URA3 GAL-NDT80::TRP1 ura3::pGPD1-GAL4(848).ER::URA3 MAM1-yeGFP::KITRP1 MTW1-tdTomato::NAT* |
| AMy27009 | diploid | *MATa dsn1::DSN1(S109D S110D)-6HIS-3FLAG::URA3 PDS1-tdTomato-KITRP1 MTW1-tdTomato::NAT MATalpha dsn1::DSN1(S109D S110D)-6HIS-3FLAG::URA3*  *PDS1-tdTomato-KITRP1 MTW1-tdTomato::NAT promURA3::TetR::GFP::LEU2,*  *tetOx224-HIS3 (Tomo’s centromeric)* |
| AMy27432 | diploid | *MATa dsn1::DSN1(S109A S110A)-6HIS-3FLAG::URA3*  *cdc20::pCLB2-3HA-CDC20::KanMX6 MATalpha dsn1::DSN1(S109A S110A)-6HIS-3FLAG::URA3 cdc20::pCLB2-3HA-CDC20::KanMX6 promURA3::TetR::GFP::LEU2 tetOx224-HIS3 (Tomo’s centromeric)* |
| AMy27647 | diploid | *MATa dsn1::DSN1(S109D S110D)-6HIS-3FLAG::URA3 cdc20::pCLB2-3HA-CDC20::KanMX6 promURA3::TetR::GFP::LEU2 tetOx224-HIS3 (Tomo’s centromeric) MATalpha dsn1::DSN1(S109D S110D)-6HIS-3FLAG::URA3 cdc20::pCLB2-3HA-CDC20::KanMX6* |
| AMy27648 | diploid | *MATa dsn1::DSN1(V104D F107D)-6HIS-3FLAG::URA3 cdc20::pCLB2-3HA-CDC20::KanMX6 promURA3::TetR::GFP::LEU2 tetOx224-HIS3 (Tomo’s centromeric) MATalpha dsn1::DSN1(V104D F107D)-6HIS-3FLAG::URA3 cdc20::pCLB2-3HA-CDC20::KanMX6* |
| AMy27773 | haploid | *MATa, dsn1::DSN1(L72A F74A)-6HIS-3FLAG::URA3 CFIII (CEN3.L.YPH983) HIS3 SUP11 (W303)* |
| AMy27774 | haploid | *MATa dsn1::DSN1(V104A F107A)-6HIS-3FLAG::URA3*  *CFIII (CEN3.L.YPH983) HIS3 SUP11* (W303) |
| AMy27775 | haploid | *MATa csm1::URA3 CFIII (CEN3.L.YPH983) HIS3 SUP11* (W303) |
| AMy27776 | haploid | *MATa csm1::URA3 CFIII (CEN3.L.YPH983) HIS3 SUP11* (W303) |
| AMy27777 | haploid | \| *MATa csm1::URA3 CFIII (CEN3.L.YPH983) HIS3 SUP11* (W303) \| \| --- \| |

**Table S3. Plasmids used in this study**

| **Plasmid #** | **Characteristics** |
| --- | --- |
| pSB1590  (AMp770) | *DSN1-6HIS-3FLAG, URA3* |
| AMp1134 | *DSN1(L72A F74A)-6HIS-3FLAG, URA3* |
| AMp1373 | *Dsn1 (L88A L92A L95A)-6HIS-3FLAG, URA3* |
| AMp1374 | *Dsn1 (E90A N94A D97A)-6HIS-3FLAG, URA3* |
| AMp1403 | *Dsn1 (L88D L92D L95D)-6HIS-3FLAG, URA3* |
| AMp1404 | *Dsn1 (E90K N94K D97K)-6HIS-3FLAG, URA3* |
| AMp1405 | *Dsn1 (V104D F107D)-6HIS-3FLAG, URA3* |
| AMp1406 | *Dsn1 (S109D S110D)-6HIS-3FLAG, URA3* |
| AMp1484 | *Dsn1 (V104A F107A)-6HIS-3FLAG, URA3* |
| AMp1591 | *Dsn1(L72A F74A V104A F107A)-6HIS-3FLAG, URA3* |
| AMp1592 | *Dsn1(L72A F74A S109A S110A)-6HIS-3FLAG, URA3* |
| AMp1640 | *Dsn1 (S109R S110R)-6HIS-3FLAG, URA3* |
| AMp1643 | *Dsn1 (L72A F74A V104A F107A S109A S110A)-6HIS-3FLAG, URA3* |
| AMp1683 | *Dsn1 (S109A S110A)-6HIS-3FLAG, URA3* |

**Table S4. Primers used for qPCR**

| **Name** | **Sequence** | **Region** |
| --- | --- | --- |
| 8214 | tgtgtatgcgttccgaactt | *CEN13 -F* |
| 8215 | ctctagccaatttatcctgtcg | *CEN13-R* |

**Table S5. Crystallographic data collection and refinement**

|  | ***Cg* Csm1^69-181^: Mam1^162-216^** | ***Cg* Csm1^69-181^: Dsn1^14-72^** | ***Cg* Csm1^69-181^: Dsn1^43-67^DD** | **His_6_-*Cg*Csm1^69-181^: *Sc* Dsn1^71-110^** |
| --- | --- | --- | --- | --- |
| **Data collection** |  |  |  |  |
| Synchrotron/Beamline | APS 24ID-E | APS 24ID-E | APS 24ID-E | SSRL 14-1 |
| Date collected | 12/5/13 | 3/7/14 | 4/22/18 | 4/15/16 |
| Resolution (Å) | 55 - 3.03 | 122 - 2.27 | 75 - 1.79 | 50 - 2.5 |
| Wavelength (Å) | 0.97918 | 0.97918 | 0.9792 | 1.127 |
| Space Group | P2_1_2_1_2_1_ | P6_1_ | H3_2_ | P2_1_ ^7^ |
| Unit Cell Dimensions (a, b, c) Å | 50.12, 58.64, 109.83 | 71.63, 71.63, 122.30 | 105.06, 105.06, 125.12 | 44.37, 203.76, 44.26 |
| Unit cell Angles (α,β,γ) ° | 90, 90, 90 | 90, 90, 120 | 90, 90, 120 | 90, 90.361, 90 |
| *I*/s (last shell) | 8.4 (1.5) | 14.3 (1.8) | 20.0 (0.9) | 18.2 (1.0) |
| ^1^*R*_sym_ (last shell) | 0.308 (1.651) | 0.116 (1.125) | 0.030 (1.440) | 0.199 (0.793) |
| ^2^*R*_meas_ (last shell) | 0.358 (1.919) | 0.128 (1.241) | 0.034 (1.629) | 0.2519 (0.938) |
| ^3^CC_1/2_ (last shell) | N/A | 0.997 (0.551) | 0.999 (0.363) | 0.996 (0.937) |
| Completeness (last shell) % | 100.0 (100.0) | 100.0 (100.0) | 99.7 (100.0) | 96.0 (76.5) |
| Number of reflections | 46342 | 93668 | 110816 | 121042 |
| *unique* | 6723 | 16495 | 25112 | 26122 |
| Multiplicity (last shell) | 6.9 (7.1) | 5.7 (5.7) | 4.4 (4.6) | 4.6 (2.8) |
| **Refinement** |  |  |  |  |
| Resolution (Å) | 55 - 3.03 | 50 - 2.27 | 52.0 – 1.79 | 50 - 2.50 |
| No. of reflections | 6675 | 32301 (16438 merged) | 47877 (25097 merged) | 25661 |
| *working* | 6360 | 30664 | 45597 | 1277 |
| *free* | 315 | 1637 | 2280 | 24384 |
| ^4^*R*_work_ (last shell) (%) | 27.12 (30.55) | 19.54 (31.13) | 19.91 (34.01) | 23.97 (39.70) |
| ^4^*R*_free_ (last shell) (%) | 28.29 (33.57) | 24.50 (34.60) | 22.32 (32.96) | 29.72 (48.82) |
| **Structure & Stereochemistry** |  |  |  |  |
| No. of atoms | 1944 | 2188 | 2190 | 4561 |
| *solvent* | 0 | 112 | 59 | 0 |
| *hydrogen* | 0 | 0 | 1051 | 0 |
| r.m.s.d. bond lengths (Å) | 0.005 | 0.007 | 0.012 | 0.007 |
| r.m.s.d. bond angles (°) | 0.763 | 0.863 | 1.18 | 0.891 |
| Ramachandran favored/allowed | 97.4/100.0 | 98.0/100.0 | 97.7/100.0 | 98.1/100.0 |
| ^5^SBGrid Data Bank ID | 607 | 608 | 609 | 610 |
| ^6^Protein Data Bank ID | 6MJ8 | 6MJB | 6MJC | 6MJE |

^1^*R*_sym_ = ∑∑_j_|*I*_j_ – 〈*I*〉|/∑*I*_j_, where *I*_j_ is the intensity measurement for reflection j and 〈*I*〉 is the mean intensity for multiply recorded reflections.

^2^*R*_meas_ = ∑_h_ [ √(*n*/(*n*-1)) ∑_j_ [*I*_hj_ - 〈*I*_h_〉] / ∑_hj_ 〈*I*_h_〉 where *I*_hj_ is a single intensity measurement for reflection h, 〈*I*_h_〉 is the average intensity measurement for multiply recorded reflections, and *n* is the number of observations of reflection h.

^3^CC_1/2_ is the Pearson correlation coefficient between the average measured intensities of two randomly-assigned half-sets of the measurements of each unique reflection (Karplus and Diederichs 2012). CC_1/2_ is considered significant above a value of ~0.15.

^4^*R*_work, free_ = ∑||F_obs_| – |F_calc_||/|F_obs_|, where the working and free *R*-factors are calculated using the working and free reflection sets, respectively.

^5^Diffraction data for each structure have been deposited with the SBGrid Data Bank (https://data.sbgrid.org) with the noted accession codes.

^6^Coordinates and structure factors for each structure have been deposited with the Protein Data Bank (http://www.pdb.org) with the noted accession codes.

^7^His_6_-*Cg*Csm1^69-181^: *Sc*Dsn1^71-110^ diffraction data could be scaled in the higher-symmetry space group P4_3_2_1_2, but final refined R-factors were significantly higher in this space group.

**Supplementary Sequences**

Archive of all sequences used to generate Fig. 1.

**Supplementary references**

Corbett KD, Harrison SC (2012) Molecular architecture of the yeast monopolin complex. Cell Rep 1:583–589. doi: 10.1016/j.celrep.2012.05.012

Crooks GE, Hon G, Chandonia J-M, Brenner SE (2004) WebLogo: a sequence logo generator. Genome research 14:1188–1190. doi: 10.1101/gr.849004

Gordon JL, Byrne KP, Wolfe KH (2011) Mechanisms of chromosome number evolution in yeast. PLoS Genet 7:e1002190. doi: 10.1371/journal.pgen.1002190

Meraldi P, McAinsh AD, Rheinbay E, Sorger PK (2006) Phylogenetic and structural analysis of centromeric DNA and kinetochore proteins. Genome biology 7:R23. doi: 10.1186/gb-2006-7-3-r23

Miller MP, Unal E, Brar GA, Amon A (2012) Meiosis I chromosome segregation is established through regulation of microtubule-kinetochore interactions. Elife 1:e00117. doi: 10.7554/eLife.00117
